# Community Pharmacist-Led Interventions to Improve Preconception and Pregnancy Health: A Systematic Review

**DOI:** 10.3390/pharmacy9040171

**Published:** 2021-10-16

**Authors:** Polly A. Scott, Ola F. Quotah, Kathryn V. Dalrymple, Sara L. White, Lucilla Poston, Jessica Farebrother, Shivali Lakhani, Marsha Alter, Mitch Blair, John Weinman, Angela C. Flynn

**Affiliations:** 1 Department of Women and Children’s Health, School of Life Course Sciences, King’s College London, London SE1 7EH, UK; p.scott1@uni.bsms.ac.uk (P.A.S.); ola.quotah@kcl.ac.uk (O.F.Q.); kathryn.dalrymple@kcl.ac.uk (K.V.D.); sara.white@kcl.ac.uk (S.L.W.); lucilla.poston@kcl.ac.uk (L.P.); 2 Human Nutrition Laboratory, Institute of Food, Nutrition and Health, ETH Zurich, 8092 Zurich, Switzerland; jessica.rigutto@hest.ethz.ch; 3 The Middlesex Pharmaceutical Group of LPCs, 1278 High Road, Whetstone, London N20 9HH, UK; shivali@middlesexpharmacy.org (S.L.); marsha@middlesexpharmacy.org (M.A.); 4 Department of Primary Care and Public Health, Imperial College London, London SW7 2AZ, UK; m.blair@imperial.ac.uk; 5 Institute of Pharmaceutical Sciences, King’s College London, London SE1 9NH, UK; john.weinman@kcl.ac.uk; 6 Department of Nutritional Sciences, School of Life Course Sciences, King’s College London, London SE1 9NH, UK

**Keywords:** community pharmacist, intervention, preconception, pregnancy, health behaviours

## Abstract

**Background**: Community pharmacist-led interventions are effective in improving health outcomes; however, their impact in improving preconception and pregnancy health is not clear. This study evaluated the effectiveness of community pharmacist-led interventions which aimed to improve health outcomes of preconception and pregnant women. **Methods:** A systematic review of the literature, consistent with PRISMA guidelines, was performed. Five electronic databases were searched up to February 2021. **Results:** Four studies, three in pregnant women and one in preconception women, were identified. The studies focused on improving micronutrient status and smoking cessation. The studies increased knowledge about, and use of, iron supplements, and improved iron status and smoking cessation rates in pregnant women, while improving knowledge regarding, and increasing the use of, preconception folic acid. The studies were ranked as weak to moderate quality. **Conclusion:** This review provides preliminary evidence for the potential benefit of community pharmacist-led interventions to improve the health of women before and during pregnancy.

## 1. Introduction

Over the last decade, the prevalence of obesity has increased [1] with significant consequences for maternal, infant and childhood outcomes. This has stimulated a public health focus to promote healthier lifestyles amongst pregnant women and those contemplating pregnancy [2,3]. It is also well recognised that sub-optimal nutrition during pregnancy can increase the risk of micronutrient deficiencies implicated in poor foetal growth and development [4]. Unhealthy dietary patterns and micronutrient deficiencies amongst pregnant women are commonly reported [5,6,7] in addition to low physical activity levels [8], smoking [9] and alcohol consumption [10].

Sub-optimal health behaviours and risk factors for adverse pregnancy outcomes are usually established well before conception. Our recent survey, which described health behaviours of over 130,000 UK women planning pregnancy, showed that 20% were smokers, less than one third took folic acid supplements and approximately half were consuming alcohol. Only half were undertaking the weekly recommended physical activity level or were consuming five portions of fruit or vegetables 4 days a week [11].

Whilst antenatal services provide point of care opportunities to deliver health behaviour advice in pregnancy, preconception care is notably absent [12]. Community settings have intrinsic, yet unmet, advantages for delivering public health initiatives to improve preconception—as well as pregnancy—health. Community pharmacists are ideally placed to inform and disseminate key health messages, which may be particularly advantageous in engaging women in areas of high deprivation. A UK study demonstrated that nearly 90% of the population can walk to a community pharmacy within 20 minutes, which increased to 100% in highly deprived areas [13].

A systematic review, conducted in 2016, showed the effectiveness of community pharmacist-led intervention in smoking cessation and the feasibility of pharmacy-delivered weight management interventions in the general population [14]. However, the impact of interventions targeting preconception and pregnant women, delivered by community pharmacists, has not been evaluated. This systematic review aimed to assess the effectiveness of community pharmacist-led interventions to improve health outcomes in women in the preconception period and/or during pregnancy. 

## 2. Materials and Methods

The systematic review was conducted in accordance with the Preferred Reporting Items for Systematic Reviews and Meta-Analyses (PRISMA) statement [15] (Supplementary Information S1). The review was registered in the PROSPERO database, registration number CRD42021232334.

### 2.1. Inclusion and Exclusion Criteria 

The inclusion and exclusion criteria were guided by the PICOS (participants, intervention, comparisons, outcomes and study design) framework, outlined in Table 1. For inclusion, studies had to meet the following criteria: (1) randomised controlled trial (RCTs) or non-RCTs (nRCTs) comparing community pharmacist-delivered interventions to inactive or usual care controls; (2) interventions targeting pregnant women or those planning pregnancy; (3) studies reporting health outcomes including weight status, physical activity, nutrition, micronutrient supplementation, smoking and alcohol status. Studies meeting the following criteria were excluded: (1) observational or qualitative research; (2) abstracts; (3) women aged less than 18 years or older than 50 years; (4) interventions delivered by pharmacists within a hospital or online; (5) studies not published in English. Studies that included pharmacists based in community pharmacies or community health centres were included. Interventions that included the involvement of the wider pharmacy team and contributions from multidisciplinary professionals were also considered. There was no restriction on study duration.

### 2.2. Literature Search

The following databases were searched up to 16/02/2021: EMBASE, MEDLINE, PsycINFO, Cumulative Index to Nursing and Allied Health Literature (CINAHL), and the Cochrane Library. The search strategy for EMBASE and MEDLINE are outlined in Supplementary Information S2. This search was limited to human studies. No limits were placed on the country of study or study date. 

### 2.3. Study Selection and Data Extraction

Following the removal of duplicates in EndNote X9, the results were exported into Rayyan, a systematic review software. Titles and abstracts were independently screened against the eligibility criteria by three reviewers (PS, OQ and AF). For studies that met the inclusion criteria, the full text articles were retrieved. Reference lists of articles were also searched to identify any eligible studies. The corresponding authors of 2 studies were contacted to confirm the role of the pharmacist [16,17]. Any disagreements between reviewers were resolved through a consensus opinion among the authors (PS, OQ and AF).

Data extraction was performed by one reviewer (PS) and independently cross checked by two reviewers (AF and OQ). The extracted data were entered into standardised tables designed for this review, with studies ordered alphabetically according to the surname of the first author.

### 2.4. Quality Assessment 

Study quality was appraised using the Effective Public Health Practice Project Quality Assessment Tool for Quantitative Studies [18]. Studies were rated using six criteria: selection bias, study design, confounders, blinding, data collection methods and withdrawals/dropouts. Studies were allocated a strong, moderate, or weak overall rating accordingly. Intervention integrity and analysis was also assessed, although this did not contribute to the overall ratings. Quality assessment was performed by two reviewers (PS and OQ). Any disagreements were again resolved through a consensus opinion among the authors (PS, OQ and AF).

### 2.5. Analysis and Synthesis 

A narrative synthesis was performed, which followed the Synthesis Without Meta-Analysis (SWiM) guidelines [19].

## 3. Results

**Figure 1:**
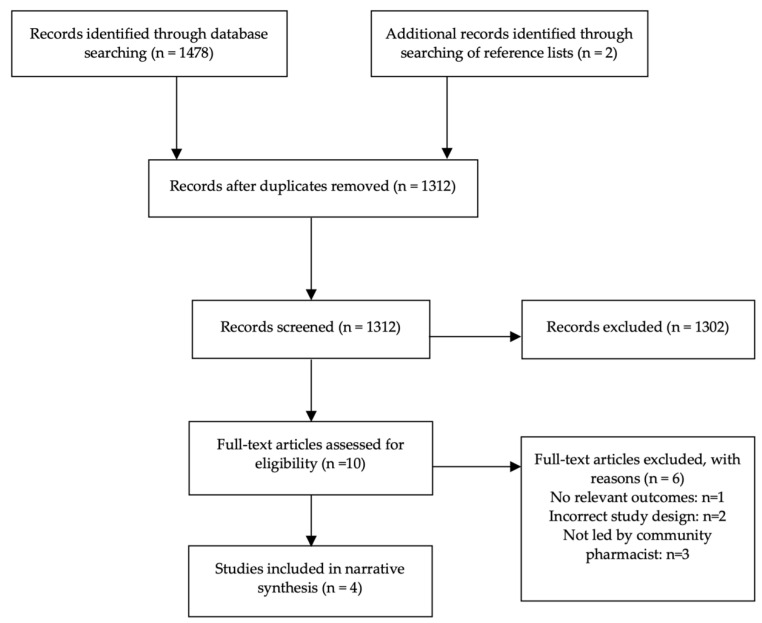
Preferred Reporting Items for Systematic Reviews and Meta-Analyses (PRISMA) flow diagram.

The study selection process is outlined in Figure 1. The literature search identified 1478 articles, and an additional two articles were identified through searching of reference lists. Following screening of titles and abstracts, ten articles were eligible for full text screening, of which four were included [20,21,22,23] and six excluded [16,17,24,25,26,27]. Reasons for exclusion included incorrect intervention setting, inappropriate study design or a target population that did not include pregnant women or those planning pregnancy.

### 3.1. Study Characteristics 

The characteristics of included studies are summarised in Table 2. These studies included a randomised pre–post control design [20], a quasi-experimental pretest–posttest control group design [23], a pilot study with randomly allocated intervention and reference pharmacies [21], and a pilot study without a control group [22]. The studies were conducted in Indonesia [20,23], the Netherlands [21] and Scotland [22], respectively. The study aims were to improve adherence to iron supplement tablets (ISTs) and improve haemoglobin (Hb) levels during pregnancy [20], improve the knowledge and use of ferrous fumarate during pregnancy [23], promote smoking cessation during pregnancy [22], and improve the knowledge and use of folic acid supplements before pregnancy [21]. The duration of the interventions varied from 30 days [20,23] to 6 months [21,22].

### 3.2. Study Population 

One study recruited women before pregnancy [21], whilst three recruited pregnant women [20,22,23], with one study specifically including women in the first and second trimesters [23]. Women were recruited through community pharmacies and community health centres. One study additionally utilised General Practitioners (GPs), antenatal clinics and libraries to provide information on the study [22]. The sample size ranged from 26 [23] to 880 women [21]. The gestational age of participants was reported in one study; within the control group, 21.9% were in the first trimester, 25% were in the second trimester and 53.1% were in the third trimester. This compared to 20.8% in the first trimester, 38.5% in the second trimester and 40.6% in the third trimester in the intervention group [20].

### 3.3. Study Interventions

All the interventions were delivered by pharmacists with support from the wider pharmacy team (Table 3). Heryadi et al. provided a single session of pharmacist-led counselling to pregnant women attending a community health centre within which a pharmacy was based. The session used a media leaflet and focused on reducing maternal anaemia through counselling on the importance of adherence to ISTs which were routinely prescribed through a government program. Participants were also advised on the correct consumption patterns of ISTs, which included information on the impact of calcium supplements, food and beverages on iron absorption [20]. 

Kusumawardani et al. also provided a single session of pharmacist-led counselling to women in the first and second trimester attending a community health centre. The counselling followed the 5A method, which aims to improve adherence to medication. This involved assessing the participants’ habits and knowledge regarding their current medication, advising the individual on the advantages and disadvantages of behaviour change, agreeing on a plan, assisting the identification of personal barriers to medication adherence, and arranging an evaluation or follow up. This aimed to improve knowledge on—and adherence to—ferrous fumarate for the prevention or treatment of maternal iron deficiency. This was the only study that referred to a specific behaviour change strategy [23].

The ‘Give It Up For Baby’ (GIUFB) programme was the basis of an antenatal smoking cessation scheme. The scheme was offered in community pharmacies within three intervention areas (area 1, area 2 and area 3) across Tayside. It involved participants attending an initial meeting with pharmacists to set a quit date, complete an interview and provide nicotine replacement therapy (NRT) if appropriate. Women attended the pharmacies every week for advice, behavioural support and carbon monoxide (CO) breath tests. Financial rewards to use in supermarkets were provided after every negative CO breath test. Participants attended weekly sessions for 12 weeks and were offered the opportunity to extend the intervention for up to 3 months postpartum [22].

In preconception women, Meijer et al. provided participants with education on folic acid supplementation. The intervention involved adding a sticker to boxes of oral contraceptives prescribed in the intervention pharmacies within the study period. The sticker included the text ‘Are you planning to have a baby? Ask for more information about folic acid in your pharmacy’. This was supplemented with an educational leaflet handed out by pharmacists alongside the prescriptions. The education aimed to inform and motivate women to ensure they took folic acid supplements whilst planning a pregnancy. The pharmacy teams were encouraged by researchers to further develop the intervention, which included trialling the use of posters, window displays, explanatory letters, ‘theme weeks’ and the provision of additional spoken information by pharmacists [21]. 

The comparison groups varied between the studies. Heryadi et al. included a control group where participants did not receive pharmacist-led counselling on adherence to ISTs and the prevention of maternal anaemia [20]. Kusumawardani et al. provided the control group with medication information as part of the normal practice at the community health centre [23]. Reference pharmacies were used by Meijer et al. [21], within which stickers and leaflets on folic acid supplements were not provided. In the work of Radley et al., the influence of the financial incentive intervention on smoking cessation rates in pregnant women was compared between the three intervention areas [22]. Although the same intervention was provided, this demonstrated the effect of intervention in areas of differing social deprivation. This study also made comparisons with data on smoking cessation rates among all pregnant women in Scotland [28]. 

### 3.4. Study Outcomes

The study outcomes are summarised in Table 4. In the report by Heryadi et al., the Hb concentration in the intervention group increased from 10.39 ± 1.24 g/dL before the intervention to 11.52 ± 0.92 g/dL post-intervention (*p* < 0.001). There was no significant change in the control group (*p* = 0.3). Between treatment groups, the Hb concentration increased in the intervention group by 8.6 times compared to the control group (odds ratio (OR) = 8.6, *p* < 0.05) after controlling for confounders. In the intervention group, 49% took 31–45 ISTs and 16.7% took 46–60 ISTs, compared to 2.1% and 1% in the control group, respectively. The calcium and IST consumption pattern improved from 0.39 ± 0.49 to 1.0 ± 0.0 in the intervention group (*p* < 0.001). Similarly, the food consumption patterns improved from 0.27 ± 0.45 to 0.03 ± 0.17 (*p* < 0.001). The calcium and food consumption patterns did not significantly change within the control group (*p* = 1) [20].

The study by Kusumawardani et al. found improvements in knowledge, perceptions, and adherence to ferrous fumarate tablets in the intervention group. Knowledge on anaemia and ferrous fumarate tablets did not differ between groups prior to intervention (*p* = 0.343) but was significantly different post intervention (*p* < 0.001, relative risk (RR) 4.54, 95% confidence interval (CI) 1.62–12.72). Similarly, adherence to ferrous fumarate tablets was not significantly different prior to intervention (*p* = 0.234), however became significant following intervention (*p* < 0.001, RR 10.29, CI 1.56–67.99). Positive and negative perceptions of taking ferrous fumarate were not significantly different between groups before intervention (*p* = 0.420). Following intervention, the perceptions were significantly different between groups (*p* < 0.001, RR 4.54, CI 1.62–12.72) [23]. 

The GIUFB programme reported quit rates of 54% at 4 weeks, 32% at 12 weeks and 17% at 3 months post-partum. Overall, 7.8% women stopped smoking in the intervention areas compared to the national average in Scotland of 3.9%. Comparisons were made between quit rates across the three areas providing the intervention, which were classified as having variable levels of social deprivation. The 47.5% 4-week quit rate in area 1 was significantly different to the 59.7% 4-week quit rate in area 2 (*p* = 0.03). The 21.5% quit rate at 3 months postpartum in area 2 was also different to the 10.1% quit rate in area 3 (*p* = 0.03) [22].

In preconception women, Meijer et al. reported that 62.5% and 20.8% of nulliparous intervention women were using or intending to use folic acid supplements, respectively, compared to 30.8% and 7.7% in the control group (*p* = 0.02). In multiparous women, 45% of the intervention group and 6.7% of the control group were aware of the correct time period to use folic acid supplements (*p* = 0.01) [21].

### 3.5. Study Quality 

The studies received global ratings from ‘moderate’ [20,23] to ‘weak’ [21,22]. This was largely due to a lack of control for confounders and lack of information on randomisation. Furthermore, two studies had a pilot study design. Pilot studies are generally underpowered to detect statistically relevant results; however, they can be used to inform on larger studies [29]. The individual ratings for the quality assessment are shown in Supplementary Information S3.

## 4. Discussion

This systematic review provides some evidence to suggest that community pharmacist-led interventions improved adherence to iron supplements, iron status and smoking cessation rates in pregnant women in addition to increasing awareness and use of folic acid supplements in women before pregnancy. However, the findings are limited by the small number and quality of the studies included.

Pharmacist-led educational counselling on the importance and use of iron supplements to reduce anaemia improved iron status in Indonesian pregnant women routinely prescribed iron supplements [20]. Similarly, pharmacist-led counselling improved knowledge on—and perceptions and use of—ferrous fumarate tablets [23]. Normal foetal growth and development is dependent upon maternal iron sufficiency during pregnancy and iron deficiency is common in pregnant women, with an estimated global prevalence of 38% [30]. Iron deficiency can lead to an increase in adverse maternal and infant outcomes [31,32]. Routine iron supplementation in pregnancy is not universal. In the UK, for example, it is recommended that women are tested for deficiency in early and late pregnancy [33]. Management of iron deficiency includes dietary advice and oral iron preparations [34]. Community-based pharmacists are ideally placed to provide advice to pregnant women on increasing dietary iron intake in addition to the optimisation of iron supplement use as part of an antenatal healthcare team.

In addition to iron supplements, Meijer et al. showed that community pharmacists improved knowledge about—and use of—folic acid supplements in women in the preconception period. In women who had a previous pregnancy, the only significant difference was increased knowledge of the correct time period to use supplements, which suggests that these women may already have had some understanding on the importance of folic acid supplements. There is a current shift in focus towards optimising health before pregnancy, with the preconception period being highlighted as critical for health across generations [3]. For example, folic acid supplementation in the preconception period can reduce neural tube defects by up to 70% [35]. We previously demonstrated poor folic acid usage in UK women planning pregnancy [11], which is consistent with other reports [36,37]. 

Public health strategies are urgently needed to increase the proportion of women taking folic acid in the preconception period. A systematic review in 2019 demonstrated uptake and acceptance of contraceptive interventions provided by community pharmacists and highlighted the opportunities to expand and integrate preconception care services including counselling on folic acid use [38]. Furthermore, a US study identified numerous opportunities in community pharmacy settings for pharmacists to provide preconception care services including screening for folic acid supplementation [26]. Together with the findings of this review, public health initiatives should consider community-based pharmacists to deliver interventions to improve folic acid knowledge and usage in preconception women.

The interventions targeting iron and folic acid supplementation were provided within one interaction with pharmacists [20,21,23]. Brief interventions are commonly used in healthcare settings, including within the ‘Making Every Contact Count’ approach, which promotes the delivery of health behaviour information within interactions with the health system [39]. The findings from this review suggest that brief interventions delivered by community-based pharmacists are feasible and lead to some improvements in health behaviours of women before and during pregnancy. However, only one study in this review referred to specific behaviour change strategies [23]. Incorporation of behaviour change strategies and training for pharmacists should be considered in future interventions. 

The intervention by Radley et al. demonstrated that pharmacist-led education and support improved smoking cessation rates in pregnant women [22]. Maternal smoking is associated with preterm birth [40], stillbirth [41] and sudden infant death syndrome [42]. Cessation of smoking in early pregnancy was shown to prevent adverse outcomes [43]. In addition, smoking cessation intervention led by community pharmacists has been shown to be effective in the general population. This was demonstrated in a meta-analysis by Brown et al., which compared intervention to active comparators (OR 1.21, 95% CI 0.86–1.71) and usual care comparators (OR 2.56, 95% CI 1.45–4.53). Smoking cessation intervention was also found to be cost effective [14]. The findings of this review support the use of community pharmacist-led smoking cessation interventions. Radley et al. used financial incentives to improve smoking cessation rates in pregnant women, which was demonstrated to be effective by a similar scheme expanded to other areas within Scotland [16] and may be a promising strategy.

Community pharmacist-led interventions were previously shown to be effective in improving health behaviours in the general population [14] and in several countries, including in the UK, there are commissioned services that focus on weight management, alcohol reduction and smoking cessation [44]. Poor health behaviours and risk factors for adverse pregnancy outcomes in pregnant and preconception women [3] suggest that services are warranted in these populations. A recent UK pilot project trained pharmacists working in local communities to provide advice to women, before and during pregnancy, on a range of topics including immunisations, early GP and antenatal appointment booking, diet and micronutrient supplementation, smoking cessation and infant feeding. Although not included in the present review, women reported finding the service useful and increased pharmacist knowledge was reported [45]. This supports the findings of the current review as it highlights the potential role that pharmacists can play in improving population health. Despite this, there has been limited research into the effectiveness of community pharmacist-led interventions targeting preconception and pregnant women. Adequately powered, large scale and robust studies are required. As community pharmacists are well positioned to deliver public health initiatives, future research priorities should focus on expanding the role of community pharmacists to improve the physical and mental health of women before and during pregnancy. Importantly, none of the included studies considered the influence of intervention on pregnancy outcomes to determine the long-term effects of improvements in health behaviours.

The strengths of this review include an extensive search of the literature with a comprehensive search strategy. Five electronic databases and reference lists were searched to identify eligible studies. The screening, data extraction and quality assessment processes were all performed in duplicate by independent reviewers. Finally, this review followed the PRISMA guidelines to ensure systematic reporting [15].

There were several limitations. The interpretation of the findings is limited by the small number and quality of the included studies. The inclusion of studies reported in English may have excluded eligible studies and introduced publication bias. The included studies were only within high- and middle-income countries, and therefore, the findings may not be generalisable to other settings. It is also important to acknowledge the focus of this review on pharmacy-based care, despite antenatal care being provided by a multidisciplinary team. Evidence of interventions targeting preconception and pregnant women within different healthcare settings was not considered within this review. 

## 5. Conclusions

Community pharmacists are well positioned to deliver public health initiatives. As demonstrated by the findings of this review, community-based pharmacists can play an important role in improving the health of women before and during pregnancy, which may lead to long term benefits to health. This systematic review found preliminary evidence that community pharmacist-led interventions improved smoking cessation rates, iron status and adherence to iron supplements in pregnancy, in addition to improved use and knowledge of folic acid supplements in women before pregnancy. However, the evidence was of a weak to moderate quality. Despite this, the initial evidence and potential benefits provide rationale for further large-scale studies to assess the effectiveness of utilising community pharmacists to improve life course health.

## Figures and Tables

**Table 1:** PICOS framework.

P—Population	Women over the age of 18 planning pregnancy or pregnant women
I—Intervention	Interventions including smoking cessation, weight management, micronutrient supplementation and alcohol reduction led by a community-based pharmacist
C—Comparison	Non-active or usual care controls
O—Outcome	Outcomes including nutritional intake, levels of physical activity, smoking and alcohol status, micronutrient supplementation and weight status
S—Study type	RCTs and nRCTs

Abbreviations: nRCT, non-randomised controlled trial; RCT, randomised controlled trial.

**Table 2:** Study characteristics of included studies.

Reference	Country of Study	Aim of Study	Study Design	Sample Size	Age (Years)
Heryadi, Sauriasari and Andrajati, 2017	Indonesia	To determine the effect of counselling by pharmacists on adherence to ISTs, IST consumption patterns and Hb levels	Randomised before–after controlled trial, single centre	192 I: n = 96 C: n = 96	I: 75 (78.1%) aged 21–35 C: 80 (83.3%) aged 21–35
Kusumawardani et al., 2019	Indonesia	To determine the influence of a brief counseling intervention by pharmacists on knowledge, perceptions, and adherence of pregnant women taking ferrous fumarate	Pretest–posttest control trial, single centre	26 I: n = 13 C: n = 13	I: 4 (31%) < 20 years, 9 (69%) 20–40 years C: 6 (46%) < 20 years, 7 (54%) 20–40 years
Meijer et al., 2005	The Netherlands	To assess the effect of information provided by community pharmacies on knowledge, attitudes and use of folic acid among women prescribed oral contraceptives	Pilot study with randomly allocated intervention and reference pharmacies, multicentre	880 I: n = 600 C: n = 280	I: mean age 33.2 years (SD 3.40) C: mean age 32.6 years (SD 3.51)
Radley et al., 2013	Scotland	To evaluate the smoking cessation outcomes and factors associated with success in pregnant women enrolled in an incentivised scheme at community pharmacies	Pilot study without a control group, multicentre	383 Intervention A1: n = 160 Intervention A2: n = 144 Intervention A3: n = 89	A1: mean age 26.7 (SD 6.10) A2: mean age 26.9 (SD 6.60) A3: mean age 27.7 (SD 5.80)

Abbreviations: A1, area 1; A2, area 2; A3, area 3; C, control group; Hb, haemoglobin; I, intervention group; IST, iron supplement tablets; n, number of individuals; SD, standard deviation.

**Table 3:** Pharmacy and intervention characteristics.

Reference	Pharmacy Characteristics	Intervention	Intervention Duration
Heryadi, Sauriasari and Andrajati, 2017	Pharmacy care provided within a community health centre in the Pagedangan Sub-district, Tangerang District	I: single session of pharmacist counselling on anaemia and ISTs with a media leaflet C: no counselling	30 days
Kusumawardani et al., 2019	Pharmacy care provided within the Jetis Community Health Center of Yogyakarta	I: single session of pharmacist counselling using the 5A model on the importance of using ferrous fumarate for anaemia treatment and prevention C: received medication information according to the practiced procedure at Jetis Community Health Center	30 days
Meijer et al., 2005	Community pharmacies in a Dutch town, randomised into groups I: 4 pharmacies C: 3 reference pharmacies	I: sticker and leaflet containing information on folic acid supplied with oral contraception C: no intervention provided	6 months
Radley et al., 2013	Community pharmacies within three intervention areas (A1, A2, A3) across Tayside	Initial meeting with pharmacist to set a quit date and provide NRT, 12 weekly visits to pharmacies for support, education and CO breath tests, financial rewards provided after every negative test, opportunity to continue to 12 weeks postpartum Comparisons made between intervention areas and national data on smoking cessation	6 months

Abbreviations: A1, area 1; A2, area 2; A3, area 3; C, control group; CO, carbon monoxide; I, intervention group; IST, iron supplement tablets; n, number of individuals; NRT, nicotine replacement therapy.

**Table 4:** Study outcomes.

Reference	Study Outcome Meaures	Study Outcomes
Heryadi, Sauriasari and Andrajati, 2017	Hb level measured with Hbocue 301 Analyzer Hb Adherence to ISTs, calcium consumption patterns, food/beverage consumption patterns measured with questionnaire	Within-group analysis: I—Hb level increased from 10.39 ± 1.24 g/dL to 11.52 ± 0.92 g/dL, *p* < 0.001 C—no changes in Hb level, *p* = 0.3Between-group analysis: I—Hb increased compared to C (OR = 8.6, *p* < 0.05) after controlling for confounders
Kusumawardani et al., 2019	Knowledge, perceptions, and adherence to ferrous fumarate assessed with questionnaires	No difference in pre-test level of knowledge, perceptions, or aderence between groups Post-test knowledge: I—0 (0.0%) low, 1 (7.7%) medium, 12 (92.3%) high C—2 (15.4%) low, 11 (84.6%) medium, 0 (0.0%) high *p* < 0.001 Post-test perceptions: I—0 (0.0%) negative, 13 (100.0%) positive C—12 (92.3%) negative, 1 (7.7%) positive *p* < 0.001 Post-test adherence: I—0 (0.0%) non-compliant, 2 (15.4%) less compliant, 11 (84.6%) compliant C—5 (38.5%) non-compliant, 7 (53.8%) less compliant, 1 (7.7%) compliant *p* < 0.001 RR values of the 3 parameters (4.55, 4.54, 10.29, respectively, with *p* < 0.05)
Meijer et al., 2005	Knowledge of folic acid, identification of information sources, planned recommendation of folic acid supplements to otherwomen, current or planned use of folic acid supplementation assessed with questionnaire	Nulligravida women: I—15 (62.5%) currently using and 5 (20.8%) intend to use folic acid supplements C—4 (30.8%) currently using and 1 (7.7%) intend to use folic acid supplements *p* = 0.02 Women with a previous pregnancy: No change in current or intended use of folic acid supplements *p* = 0.42. I—9 (45.0%) knew the correct time period to take folic acid C—1 (6.7%) knew the correct time period to take folic acid *p* = 0.01
Radley et al., 2013	Short and long term quit rates measured with CO breath testsEngagement with programme measured with attendance	Quit rates were 54% at 4 weeks, 32% at 12 weeks and 17% at 3 months post partum 7.8% of women in the intervention areas compared to 3.9% of all pregnant smokers in Scotland quit at 4 weeks By intervention areas: A1—76 (47.5%) quit rate at 4 weeks A2—86 (59.7%) quit rate at 4 weeks *p* = 0.0330 A2—31 (21.5%) quit rate at 3 months postpartum A3—9 (10.1%) quit rate at 3 months postpartum *p* = 0.0248

Abbreviations: A1, area 1; A2, area 2; A3, area 3; CO, carbon monoxide; CI, confidence interval; C, control group; Hb, haemoglobin; I, intervention group; IST, iron supplement tablets; OR, odds ratio; RR, relative risk. Statistical significance if *p* < 0.05.

## Supplementary Materials

The following are available online at https://www.mdpi.com/article/10.3390/pharmacy9040171/s1, Supplementary Information S1: PRISMA checklist; Supplementary Information S2: Full search strategy for the MEDLINE and EMBASE databases; Supplementary Information S3: Quality assessment for studies included in the systematic review.

## References

[1] World Health Organization Obesity and Overweight. https://www.who.int/news-room/fact-sheets/detail/obesity-and-overweight.

[2] Poston L., Caleyachetty R., Cnattingius S., Corvalan C., Uauy R., Herring S., Gillman M.W. (2016). Preconceptional and maternal obesity: Epidemiology and health consequences. Lancet Diabetes Endocrinol..

[3] Stephenson J., Heslehurst N., Hall J., Schoenaker D.A., Hutchinson J., Cade J.E., Poston L., Barrett G., Crozier S.R., Barker M. (2018). Before the beginning: Nutrition and lifestyle in the preconception period and its im-portance for future health. Lancet.

[4] Gernand A.D., Schulze K.J., Stewart C.P., West K.P., Christian P. (2016). Micronutrient deficiencies in pregnancy worldwide: Health effects and prevention. Nat. Rev. Endocrinol..

[5] Farebrother J., Dalrymple K.V., White S.L., Gill C., Brockbank A., Lazarus J.H., Godfrey K.M., Poston L., Flynn A.C. (2021). Iodine status of pregnant women with obesity from inner city populations in the United Kingdom. Eur. J. Clin. Nutr..

[6] Blumfield M.L., Hure A., MacDonald-Wicks L., Smith R., Collins C.E. (2012). Systematic review and meta-analysis of energy and macronutrient intakes during pregnancy in developed countries. Nutr. Rev..

[7] Flynn A.C., Seed P.T., Patel N., Barr S., Bell R., Briley A.L., Godfrey K.M., Nelson S.M., Oteng-Ntim E. (2016). Dietary patterns in obese pregnant women; influence of a behavioral intervention of diet and physical activity in the UPBEAT randomized controlled trial. Int. J. Behav. Nutr. Phys. Act..

[8] Nascimento S.L., Surita F.G., Godoy A.C., Kasawara K.T., Morais S.S. (2015). Physical activity patterns and fac-tors related to exercise during pregnancy: A cross sectional study. PLoS ONE.

[9] Public Health England, Local Tobacco Control Profiles. https://fingertips.phe.org.uk/search/delivery.

[10] Mårdby A.-C., Lupattelli A., Hensing G., Nordeng H. (2017). Consumption of alcohol during pregnancy—A multinational European study. Women Birth.

[11] McDougall B., Kavanagh K., Stephenson J., Poston L., Flynn A.C., White S.L. (2021). Health behaviours in 131,182 UK women planning pregnancy. BMC Pregnancy Childbirth.

[12] Public Health England, Making the Case for Preconception Care. https://www.gov.uk/government/publications/preconceptioncare-making-the-case.

[13] Todd A., Copeland A., Husband A., Kasim A., Bambra C. (2014). The positive pharmacy care law: An area-level analysis of the relationship between community pharmacy distribution, urbanity and social deprivation in England. BMJ Open.

[14] Brown T.J., Todd A., O’Malley C., Moore H., Husband A., Bambra C., Kasim A.S., Sniehotta F., Steed L., Smith S. (2016). Community pharmacy-delivered interventions for public health priorities: A systematic review of interventions for alcohol reduction, smoking cessation and weight management, including meta-analysis for smoking cessation. BMJ Open.

[15] Moher D., Liberati A., Tetzlaff J., Altman D.G., The PRISMA Group (2009). Preferred reporting items for systematic reviews and meta-analyses: The PRISMA statement. PLoS Med..

[16] Tappin D., Bauld L., Purves D., Boyd K., Sinclair L., MacAskill S., McKell J., Friel B., McConnachie A., De Caestecker L. (2015). Financial incentives for smoking cessation in pregnancy: Randomised controlled trial. BMJ.

[17] Forinash A.B., Yancey A., Chamness D., Koerner J., Inteso C., Miller C., Gross G., Mathews K. (2018). Smoking Cessation Following Text Message Intervention in Pregnant Women. Ann. Pharmacother..

[18] Effective Public Health Practice Project Quality Assessment Tool for Quantitative Studies. http://www.ephpp.ca/tools.html.

[19] Campbell M., McKenzie J.E., Sowden A., Katikireddi S.V., Brennan S.E., Ellis S., Hartmann-Boyce J., Ryan R., Shepperd S., Thomas J. (2020). Synthesis without meta-analysis (SWiM) in systematic reviews: Reporting guideline. BMJ.

[20] Heryadi P.L., Sauriasari R., Andrajati R. (2017). The influence of pharmacist counseling on changes in hemoglobin levels of pregnant women at a community health center in Indonesia. Asian J. Pharm. Clin. Res..

[21] Meijer W., de Smit D.J., Jurgens R.A., De Jong-Van Den Berg L.T.W. (2010). Improved periconceptional use of folic acid after patient education in pharmacies: Promising results of a pilot study in the Netherlands. Int. J. Pharm. Pr..

[22] Radley A., Ballard P., Eadie D., MacAskill S., Donnelly L., Tappin D. (2013). Give it up for baby: Outcomes and fac-tors influencing uptake of a pilot smoking cessation incentive scheme for pregnant women. BMC Public Health.

[23] Kusumawardani N., Darmawan E., Akrom A., Retnowati S. (2019). Brief counseling by pharmacists enhances the knowledge, perceptions, and compliance of first and second-trimester pregnant women consuming ferrous fumarate at Jetis Community Health Center of Yogyakarta. Pharmaciana.

[24] Foisy M.M., Akai P.S. (2004). Pharmaceutical Care for HIV patients on directly observed therapy. Ann. Pharmacother..

[25] Forinash A.B., Yancey A.M., Shyken J. (2020). Pharmacist lead vaccination rates in opioid addicted obstetric patients. J. Am. Coll. Clin. Pharm..

[26] Luli A.J., Tran N., Ataya A., Rafie S. (2020). Patient screenings for preconception health interventions at a community pharmacy. Pharmacy.

[27] Mardle T., Merrett S., Wright J., Percival F., Lockhart I. (2012). Real world evaluation of three models of NHS smoking cessation service in England. BMC Res. Notes.

[28] Tappin D.M., Macaskill S., Bauld L., Eadie U., Shipton D., Galbraith L. (2010). Smoking prevalence and smoking cessation services for pregnant women in Scotland. Subst. Abus. Treat. Prev. Policy.

[29] Kistin C., Silverstein M. (2015). Pilot studies: A critical but potentially misused component of interventional re-search. JAMA.

[30] Stevens G.A., Finucane M.M., De-Regil L.M., Paciorek C.J., Flaxman S.R., Branca F., Peña-Rosas J.P., Bhutta Z.A., Ezzati M. (2013). Global, regional, and national trends in haemoglobin concentration and prevalence of total and severe anaemia in children and pregnant and non-pregnant women for 1995–2011: A systematic analysis of population-representative data. Lancet Glob. Health.

[31] Mahmood T., Rehman A.U., Tserenpil G., Siddiqui F., Ahmed M., Siraj F., Kumar B. (2019). The association be-tween iron-deficiency anemia and adverse pregnancy outcomes: A retrospective report from Pakistan. Cureus.

[32] Kumar K.J., Asha N., Murthy D.S., Sujatha M., Manjunath V. (2013). Maternal anemia in various trimesters and its effect on newborn weight and maturity: An observational study. Int. J. Prev. Med..

[33] National Institute for Health and Care Excellence Antenatal Care for Uncomplicated Pregnancies. https://www.nice.org.uk/guidance/cg62/chapter/1-Guidance.

[34] Pavord S., Myers B., Robinson S., Allard S., Strong J., Oppenheimer C. (2012). UK guidelines on the management of iron deficiency in pregnancy. Br. J. Haematol..

[35] De-Regil L.M., Peña-Rosas J.P., Fernández-Gaxiola A.C., Rayco-Solon P. (2015). Effects and safety of periconceptional oral folate supplementation for preventing birth defects. Cochrane Database Syst. Rev..

[36] Ishitsuka K., Sasaki S., Yamamoto-Hanada K., Mezawa H., Konishi M., Ohya Y. (2020). Changes in dietary intake in pregnant women from periconception to pregnancy in the japan environment and children’s study: A Nationwide Japanese Birth Cohort Study. Matern. Child Health J..

[37] Bitzer J., von Stenglin A., Bannemerschult R. (2013). Women’s awareness and periconceptional use of folic acid: Data from a large European survey. Int. J. Women’s Health.

[38] Bright D.R., DiPietro Mager N.A. (2019). Preconception care and contraception services: Opportunities for community pharmacists. J Am. Coll. Clin. Pharm..

[39] NHS Health Education England Making Every Contact Count. http://www.makingeverycontactcount.co.uk/.

[40] Ko T.J., Tsai L.Y., Chu L.C., Yeh S.J., Leung C., Chen C.Y., Chou H.C., Tsao P.N., Chen P.C., Hsieh W.S. (2014). Parental smoking during pregnancy and its association with low birth weight, small for gestational age, and pre-term birth offspring: A birth cohort study. Pediatr. Neonatol..

[41] Marufu T.C., Ahankari A., Coleman T., Lewis S. (2015). Maternal smoking and the risk of still birth: Systematic review and meta-analysis. BMC Public Health.

[42] Zhang K., Wang X. (2013). Maternal smoking and increased risk of sudden infant death syndrome: A meta-analysis. Leg. Med..

[43] McCowan L.M.E., Dekker G.A., Chan E., Stewart A., Chappell L.C., Hunter M., Moss-Morris R., North R.A. (2009). On behalf of the SCOPE consortium Spontaneous preterm birth and small for gestational age infants in women who stop smoking early in pregnancy: Prospective cohort study. BMJ.

[44] Pharmaceutical Services Negotiating Committee, Community Pharmacy Contractual Framework—A Summary. https://psnc.org.uk/contract-it/the-pharmacy-contract/.

[45] Pharmacy4Mums2B—Final Evaluation Report. https://www.middlesexlpcs.org.uk/wp-content/uploads/2020/07/P4M2B-v10-FINAL-200819-PDF.pdf.

