# Supplementary material for: Community Pharmacist-Led Interventions to Improve Preconception and Pregnancy Health: A Systematic Review
Source: Pharmacy (Basel). 2021 Oct 16;9(4):171. doi: 10.3390/pharmacy9040171 (PMC8544701; doi:10.3390/pharmacy9040171)
Supplement: Supplementary file 1 [file pharmacy-09-00171-s001.zip › pharmacy-1417841-supplementary.pdf]

Supplementary Information

S1: PRISMA checklist

Section/topic	#	Checklist item	Reported on page #
**TITLE**			
Title	1	Identify the report as a systematic review, meta-analysis, or both.	1
**ABSTRACT**			
Structured summary	2	Provide a structured summary including, as applicable: background; objectives; data sources; study eligibility criteria, participants, and interventions; study appraisal and synthesis methods; results; limitations; conclusions and implications of key findings; systematic review registration number.	1
**INTRODUCTION**			
Rationale	3	Describe the rationale for the review in the context of what is already known.	1-2
Objectives	4	Provide an explicit statement of questions being addressed with reference to participants, interventions, comparisons, outcomes, and study design (PICOS).	2
**METHODS**			
Protocol and registration	5	Indicate if a review protocol exists, if and where it can be accessed (e.g., Web address), and, if available, provide registration information including registration number.	2
Eligibility criteria	6	Specify study characteristics (e.g., PICOS, length of follow-up) and report characteristics (e.g., years considered, language, publication status) used as criteria for eligibility, giving rationale.	2
Information sources	7	Describe all information sources (e.g., databases with dates of coverage, contact with study authors to identify additional studies) in the search and date last searched.	2-3
Search	8	Present full electronic search strategy for at least one database, including any limits used, such that it could be repeated.	2
Study selection	9	State the process for selecting studies (i.e., screening, eligibility, included in systematic review, and, if applicable, included in the meta-analysis).	3

Section/topic	#	Checklist item	Reported on page #
Data collection process	10	Describe method of data extraction from reports (e.g., piloted forms, independently, in duplicate) and any processes for obtaining and confirming data from investigators.	3
Data items	11	List and define all variables for which data were sought (e.g., PICOS, funding sources) and any assumptions and simplifications made.	2-3
Risk of bias in individual studies	12	Describe methods used for assessing risk of bias of individual studies (including specification of whether this was done at the study or outcome level), and how this information is to be used in any data synthesis.	3
Summary measures	13	State the principal summary measures (e.g., risk ratio, difference in means).	N/A
Synthesis of results	14	Describe the methods of handling data and combining results of studies, if done, including measures of consistency (e.g., I^2) for each meta-analysis.	N/A
Risk of bias across studies	15	Specify any assessment of risk of bias that may affect the cumulative evidence (e.g., publication bias, selective reporting within studies).	N/A
Additional analyses	16	Describe methods of additional analyses (e.g., sensitivity or subgroup analyses, meta-regression), if done, indicating which were pre-specified.	N/A
**RESULTS**			
Study selection	17	Give numbers of studies screened, assessed for eligibility, and included in the review, with reasons for exclusions at each stage, ideally with a flow diagram.	4
Study characteristics	18	For each study, present characteristics for which data were extracted (e.g., study size, PICOS, follow-up period) and provide the citations.	4-9
Risk of bias within studies	19	Present data on risk of bias of each study and, if available, any outcome-level assessment (see Item 12).	9
Results of individual studies	20	For all outcomes considered (benefits or harms), present, for each study: (a) simple summary data for each intervention group and (b) effect estimates and confidence intervals, ideally with a forest plot.	7-9
Synthesis of results	21	Present results of each meta-analysis done, including confidence intervals and measures of consistency.	N/A
Risk of bias across studies	22	Present results of any assessment of risk of bias across studies (see Item 15).	N/A

Section/topic	#	Checklist item	Reported on page #
Additional analysis	23	Give results of additional analyses, if done (e.g., sensitivity or subgroup analyses, meta-regression [see Item 16]).	N/A
**DISCUSSION**			
Summary of evidence	24	Summarize the main findings including the strength of evidence for each main outcome; consider their relevance to key groups (e.g., health care providers, users, and policy makers).	9-11
Limitations	25	Discuss limitations at study and outcome level (e.g., risk of bias), and at review level (e.g., incomplete retrieval of identified research, reporting bias).	9-11
Conclusions	26	Provide a general interpretation of the results in the context of other evidence, and implications for future research.	11
**FUNDING**			
Funding	27	Describe sources of funding for the systematic review and other support (e.g., supply of data); role of funders for the systematic review.	11

S2: Full search strategy for the MEDLINE and EMBASE databases

1 exp Community Pharmacy Services/
2 Pharmacies/
3 exp Pharmacists/
4 exp Pharmacists' Aides/
5 Pharmacy/
6 chemist.tw.
7 (communit$ adj7 pharmac$).tw.
8 (office$ adj7 pharmacy$).tw.
9 ((pharmacy or pharmacist? or pharmacies) adj3 (community or counsel$ or advice or care)).tw.
10 (pharmacist? adj3 (front line or 'one to one' or face to face)).tw.
11 (pharmacist? or pharmacy or pharmacies).tw.
12 ((pharmacist? or pharmacy) adj3 (aide or aides or assistant? or staff)).tw.
13 (Pharmacist? adj2 (care or delivered)).tw.
14 (pharmacist? adj3 (counsel$ or (patient? adj2 education$) or led or intervention? or public health or diagnos$)).tw.
15 or/1–14
16 exp Obesity/
17 exp Body Weight/
18 exp Body Weight Changes/
19 exp Weight Gain/ or exp Weight Loss/
20 (obese or obesity).tw.
21 overweight.tw.
22 weight.tw.
23 diet$.tw.
24 nutrition$.tw.
25 (physical$ adj activ$).tw.
26 exercise$.tw.
27 lifestyle$.tw.
28 (bmi$ or (body adj mass ind$)).tw.
29 (waist adj6 circumference$).tw.
30 ((weight adj2 (control or reduction) adj2 (advice or counsel$ or program$ or intervention?)) or (weight adj manag$)).tw.

31 ((overweight or obese or obesity) adj4 (Advice or counsel$ or intervention? or program$)).tw.

32 or/16–31

33 exp Smoking/ or exp Smoking Cessation/

34 nicotine.tw.

35 cigarette$.tw.

36 (nicotine replacement therapy or NRT).tw.

37 smoking cessation.tw.

38 smok$.tw.

39 exp "Tobacco Use Cessation"/

40 exp Smoking Cessation/

41 (smoking cessation or (quit$ adj2 smok$)).tw.

42 ((reduce or reducing) adj3 ('tobacco use' or cigarette? or smoking or addiction)).tw.

43 or/33–42

44 alcohol.mp.

45 exp Alcohols/

46 exp Alcohol Drinking/

47 exp Alcoholism/

48 exp Drinking Behavior/

49 (drink$).tw.

50 beer.tw.

51 wine.tw.

52 ethanol.tw.

53 drunk.tw.

54 (addict$ or (alcohol adj2 (abus$ or misus$))).tw.

55 alcohol$.tw.

56 drunk$.tw.

57 intoxicat$.tw.

58 or/44–57

59 Pregnancy/

60 pregnan*.tw.

61 Gravidity/

62 gravid*.tw.

63	gestation*.tw.
64	Pregnant Women/
65	pregnant wom#n.tw.
66	Prepregnan/
67	Prepregnan*.tw.
68	Preconception/
69	Preconception*.tw.
70	(child adj3 bearing).tw.
71	childbearing.tw.
72	matern*.tw.
73	or/59-72
74	32 or 43 or 58 or 73
75	(animals not humans).mp.
76	74 not 75
77	15 and 75
78	limit 77 to humans

S3: Quality assessment for studies included in the systematic review

	Quality assessment criteria						
Reference	Selection bias	Study design	Confounders	Blinding	Data collection methods	Withdrawals and dropouts	Global rating
Heryadi *et al.,* 2017	Moderate	Strong	Strong	Moderate	Strong	Weak	Moderate
Kusumawardani *et al.,* 2019	Weak	Strong	Strong	Moderate	Strong	Strong	Moderate
Meijer *et al.,* 2005	Moderate	Strong	Weak	Moderate	Weak	Moderate	Weak
Radley *et al.,* 2013	Weak	Weak	Weak	Moderate	Strong	Weak	Weak
